# Contemporary Management of Prostate Cancer

**DOI:** 10.12688/f1000research.7183.1

**Published:** 2016-02-16

**Authors:** Katherine Cotter, Badrinath Konety, Maria A. Ordonez

**Affiliations:** 1 Department of Urology, University of Minnesota, Minneapolis, Minnesota, 55455, USA

**Keywords:** prostate, cancer, tumor

## Abstract

Prostate cancer represents a spectrum ranging from low-grade, localized tumors to devastating metastatic disease. We discuss the general options for treatment and recent developments in the field.

Prostate cancer is the most common non-cutaneous malignancy in American men and the second most common cause of cancer-specific mortality. In 2015, prostate cancer will be diagnosed in an estimated 220,800 men in the United States, and an estimated 27,540 men will die of the disease ( http://www.cancer.gov/cancertopics/types/prostate) ^1^. Determining which of these cancers are likely to progress remains a significant challenge in management. Novel translational research has helped to guide these decisions to maximize oncologic outcomes while minimizing morbidity of overtreatment. Integration of tumor biology with clinical practice may lead to a more individualized, patient-specific treatment plan.

Additionally, novel treatment options aim to achieve a higher therapeutic index. This article reviews the general management of both localized and metastatic prostate cancer, with a focus on emerging research aimed at guiding both management decisions and developments in targeted therapies.

Risk stratification of clinically localized prostate cancer has served as a guide to counsel patients on treatment options ( Table 1) ^2, 3^. The American Urological Association (AUA) guidelines offer active surveillance (AS), radical prostatectomy (RP) with or without pelvic lymphadenectomy, external beam radiotherapy (EBRT), and interstitial radiotherapy/brachytherapy for clinically localized disease (T1, T2), with an impetus toward treatment with higher risk tumors ^4^. Limited evidence has led to a lack of consensus regarding the preferred treatment.

**Table 1.** Risk stratification of clinically localized prostate cancer.

	PSA (ng/mL)	Gleason score	Clinical stage
**Low risk**	<10	≤6	cT1 or T2a
**Intermediate risk**	≥10 to <20	7	cT2b
**High risk**	≥20	≥8	cT2c or T3

Adapted from 2.

## Prostate cancer detection

Due to a shift in disease stage at presentation, the proportion of patients presenting with high-risk or metastatic disease has declined, as have the death rates. In 1990, the 5-year relative survival rate of prostate cancer was 88.4%, while in 2007, 5-year survival was 99.7% (Surveillance, Epidemiology, and End Results [SEER] Medicare Cancer Statistics, 2007–2011); this demonstrates a potential benefit from early detection. However, aggressive screening and superior modes of detection carry the risk of overtreatment. Analysis of greater than 10,000 men in the Cancer of the Prostate Strategic Urologic Research Endeavor (CaPSURE) registry in 2007 showed a significant increase in clinical T1c disease and a transition toward greater use of AS. The CaPSURE registry consists of patients from a total of 31 U.S. centers with biopsy-proven prostate adenocarcinoma. From 2000–2006, just over 50% of all new prostate cancer diagnoses within the registry were low risk (prostate-specific antigen [PSA] <10 ng/mL, Gleason score ≤6, and clinical stage ≤Ta). Within this group of patients, parameters such as percentage of biopsy cores positive and stratification of the individual Gleason scores ^5^ have subdivided this population further. This study demonstrated a significant shift toward low-risk characteristics and a potential under-use of AS as an option. Therefore, use of the Cancer of the Prostate Risk Assessment (CAPRA) score, a risk stratification tool, has allowed substratification even within a low-risk population to predict biochemical recurrence and counsel patients more effectively ^6^.

Advances in imaging have also improved the detection of prostate cancer. Multi-parametric magnetic resonance imaging (mp-MRI) ^7^ incorporates functional parameters to T2-weighted imaging, providing dynamic imaging of prostate lesions. A recently published prospective cohort study compared standard 12-core ultrasound-guided biopsy with targeted, MRI-guided fusion biopsy in over 1000 men with at least one prior negative biopsy from 2007–2014. Each patient had an mp-MRI, and those with suspicious lesions underwent fusion biopsy, followed by a standard ultrasound-guided biopsy by another urologist unaware of the MRI results. Results showed that fusion biopsy was able to detect high-grade prostate cancer with higher sensitivity than standard biopsy alone (77% versus 53%, respectively); however, fusion biopsy demonstrated lower sensitivity in detecting low-grade disease. The long-term clinical significance remains to be shown but points to improved detection of clinically significant prostate cancer ^8^.

In an effort to further risk stratify prostate cancer patients and find markers for aggressive disease, genomic biomarkers have been integrated into clinical practice and include Prolaris (Myriad Genetics), Oncotype DX Prostate Cancer Assay (Genomics Health, Inc), transmembrane protease, serine 2 (TMPRSS-2), and prostate cancer antigen 3 (PCA3) ^7^. Oncotype DX tests for specific gene expression in prostate biopsy tissue and, in conjunction with National Comprehensive Cancer Network (NCCN) risk criteria, can be used to determine candidacy for AS ^9^. The Prolaris test assesses the expression of genes primarily involved in cell cycle progression to directly evaluate tumor growth and determine the likelihood of disease progression ^10^. While further studies are warranted, such biomarkers have been a promising area of investigation.

Recent research has investigated the application of kallikrein-based tests to supplement PSA testing. Integration of kallikrein markers, which include free PSA (fPSA), single-chain intact PSA (iPSA), total PSA (tPSA), and human kallikrein 2 (hK2), has shown increased specificity in predictive models at PSA 2–10 ng/mL. The potential value of these markers would be of greatest use for patients with PSA of 2–10 ng/ml and low PSA density based on digital rectal examination ^11^.

In addition, proPSA, a molecular, inactive precursor of PSA, has also been posited as a potential marker, specifically the truncated (-2) form. hK2, a kallikrein-related peptidase, cleaves proPSA into the active form. Patients with prostate cancer may demonstrate elevated levels of proPSA than patients without cancer ^12^. The prostate health index (PHI) combines tPSA, % fPSA, and (-2) proPSA. Further areas of investigation include prospective studies and applicability in patients with a strong family history of prostate cancer as well as those on 5-α-reductase inhibitors ^13^.

Newer diagnostic imaging modalities used mainly to detect disease recurrence after definitive therapy include prostate-specific membrane antigen based positron emission tomography (PSMA PET), C ^11^ choline PET, and sodium fluoride bone scan. A recent prospective analysis of 38 men with biochemical recurrence (mean PSA 1.74 ng/mL) following either RP or EBRT showed that PSMA PET demonstrated greater sensitivity than a standard (18)F-fluoromethylcholine PET to detect disease ^14^. As the PSA window for salvage therapy is often lower than the threshold reliably detected by standard PET, such imaging options may allow patients to have treatment at an earlier stage. Retrospective analysis of C ^11^ PET has been predictive of positive findings for recurrence with a PSA of 1.24 ng/mL or PSA velocity of 1.32 ng/mL/year ^15^. Both fluorocholine and sodium fluoride PET computed tomography (CT) bone scans showed ability to detect bony metastases specifically, when studied in a prospective series of 42 prostate cancer patients with a minimum 6-month follow-up period ^16^.

## Treatment

According to the AUA guidelines regarding the management of clinically localized prostate cancer, each patient should be informed about the risks and benefits of available initial interventions, including AS, EBRT, brachytherapy, and RP ^3^.

One randomized clinical trial showed a reduction in overall mortality with RP versus watchful waiting ^17^, as well as a reduction in disease-specific death, local progression, and metastasis. The Prostate Cancer Intervention versus Observation Trial (PIVOT) trial, which compared RP to AS, enrolled a more contemporary, screen-detected cohort of men and showed a significant benefit in overall survival with RP only in patients with pre-treatment PSA >10 ng/ml or high-risk disease ^18^, but no significant difference in patients with low-risk cancer. These findings support the role of AS as an option in patients with low-volume, low-grade disease. However, the question frequently remains which patients best qualify for AS. Novel tools, such as prostate MRI and serum and urinary biomarkers, aim to provide a more accurate method of determining which patients are appropriate for AS ^19^. Cost may prove a limiting factor in the widespread use of these tools but, when used in conjunction with PSA, Gleason score, and clinical stage, may translate to more informed patient decision making.

## Radiation therapy

EBRT and brachytherapy may be offered as monotherapy or in conjunction depending on patient and tumor factors; hormone therapy may also play a synergistic role in patients with intermediate- and high-risk disease by promoting cellular apoptosis ^3^. A 6-month course of neoadjuvant hormonal therapy has demonstrated a survival benefit in intermediate-risk patients ultimately receiving EBRT, and adjuvant hormonal therapy has been shown to prolong survival in high-risk patients and/or patients with locally advanced disease ^20, 21^, particularly with a 3-year course of hormonal therapy after EBRT ^22, 23^. Hence, combined androgen deprivation therapy (ADT) and EBRT should be discussed and offered to patients with locally advanced prostate cancer. Two randomized controlled clinical trials demonstrated that high-dose radiation may reduce the risk of PSA recurrence ^24^. Methods to target tumors with increasing accuracy, including the advent of intensity-modulated radiotherapy, have led to greater dose escalation of radiation with a wider margin of safety ^25^.

Primary hormonal monotherapy may be offered in the setting of limited life expectancy and in patients unable to proceed with other local therapies ^26^. Benefits of treatment, however, must be weighed against potential complications impacting the patient’s quality of life, including hot flashes, truncal obesity, increased risk of metabolic syndrome, and cardiovascular side effects (particularly in men with pre-existing cardiac disease). Additional treatment options for localized prostate cancer include cryotherapy and high-intensity focused ultrasound (HIFU), neither of which are currently recommended first-line treatments in the United States.

## Alternate therapies

In addition to the standard treatment options outlined above, prostate cryotherapy and HIFU are alternate therapies that are less widely available for the treatment of localized prostate cancer. A review of cryotherapy outcomes showed 5-year biochemical survival rates of approximately 70% for patients undergoing whole gland ablation. Patients in this group were also found to have higher rates of erectile dysfunction ^27^. Similarly, HIFU therapy for prostate cancer demonstrated 5- and 10-year biochemical survival rates of 80% and 61%. Potency was preserved in 25% of patients after treatment. Improvements in imaging, such as mp-MRI, aim to optimize the therapeutic index of focal therapy. This burgeoning field couples mp-MRI with ablative techniques to treat index prostate lesions. Furthermore, mp-MRI has allowed for more accurate follow-up after definitive focal therapy. Cryotherapy and brachytherapy, as well as HIFU (in Europe), are established focal therapy modalities; however, newer techniques, such as irreversible electroporation (Nanoknife™) ^28^ and laser interstitial therapy, are under current investigation and may demonstrate promise.

## Disease recurrence after local therapy

In the majority of patients, RP is curative; however, data suggests that within 10 years after surgery, up to 33% of men will have evidence of recurrent disease ^29^. The risk is greater in patients with adverse surgical pathologic features, including extraprostatic extension, positive surgical margins, and seminal vesicle invasion ^30^. Adjuvant radiation may be offered to these patients and has been shown to reduce the risk of local and PSA recurrence as well as clinical progression ^31^.

Biochemical recurrence after prostatectomy is defined as a PSA of ≥0.2 ng/mL on two separate tests. Data indicates that the salvage radiotherapy is most effective when administered with a low PSA, i.e. 0.5 to 1.5 ng/mL ^32^. Options include surveillance, salvage radiation, ADT, and enrolment in clinical trials. In the post-radiation setting, biochemical failure is defined as PSA ≥2.0 ng/mL over the nadir, or three consecutive rises ^33^. Options include surveillance, salvage prostatectomy, ADT, cryotherapy, and clinical trials.

Pre-treatment nomograms can be used to determine an individual patient’s risk of specific clinical endpoints and therefore may supplement the patient’s discussion about the need for multimodal therapy. Specifically, pre-prostatectomy nomograms predict the risk of adverse pathologic features, as well as of disease recurrence. Models, such as the Kattan nomogram and University of California, San Francisco (UCSF) CAPRA score ^34^, use pre-treatment PSA, biopsy results, clinical stage, and other factors to estimate certain outcomes.

The D’Amico classification uses PSA, Gleason score, and clinical stage to risk stratify patients into low-, intermediate-, and high-risk categories ^2^. Other models, such as the UCSF-CAPRA score, stratify estimated risk using a 0–10 numeric scale calculated with patient information including age and PSA at diagnosis, Gleason score, clinical stage, and percent of biopsy cores involved ^34^. Nomograms can provide patients and physicians with objective information to select treatment plans and estimate risk.

When biochemical recurrence has occurred, PSA kinetics can be used to calculate the risk of local versus distant recurrence, as well as to guide indications for obtaining bone scans and other imaging studies. Patients with a PSA doubling time (PSADT) of >15 months have a low cancer-specific mortality rate at 10 years and therefore may be candidates for AS, particularly if life expectancy is <10 years ^35^. Conversely, a PSADT of 3 months or under suggests distant metastatic disease and a median 6-year survival.

## Advanced disease

Locally advanced and distant metastatic disease frequently require a multimodal treatment approach. For locally advanced prostate cancer, main treatment options include EBRT with interstitial radiotherapy, RP with hormonal therapy, and EBRT with hormonal therapy, as discussed above. In the setting of PSA rise post-treatment, time to PSA recurrence, PSADT, and Gleason score may be predictive of progression to metastatic disease ^36^. Therefore, these parameters may be used to determine which therapies are best suited for the patient given the likelihood of disease progression.

Options for advanced systemic prostate cancer with the aim to achieve castrate-levels of testosterone include bilateral orchiectomy (surgical castration), luteinizing hormone receptor analogs with or without complete androgen blockade, androgen receptor (AR) antagonists (steroidal or non-steroidal), and ketoconazole with steroids. Future directions in the field of prostate cancer management include RP for advanced and oligometastatic disease in the context of combined modality therapy. Patients with metastatic castration-resistant prostate cancer (mCRPC) may be candidates for chemotherapy or immunotherapy depending on prior therapies received, presence and severity of symptoms, documented metastases on imaging, and performance status ^3^. Sipuleucel-T, an autologous cellular immunotherapy, is an option for men with good performance status, no prior docetaxel therapy or visceral metastases ^37^, and symptoms from metastases not requiring narcotic medication. The Immunotherapy for Prostate Adenocarcinoma Treatment (IMPACT) trial demonstrated a 4.1-month survival advantage with sipuleucel-T versus placebo, although no effect on time to disease progression was found ^38^. Cabazitaxel, a tubulin-binding taxane, may be offered in the post-docetaxel setting and was shown to have greater overall (15.1 versus 12.7 months, *p* <0.0001) and progression-free survival (2.8 versus 1.4 months, *p* <0.0001) when compared to mitoxantrone ^39^. Abiraterone is an androgen biosynthesis inhibitor shown to prolong survival in men with mCRPC after receiving chemotherapy. Enzalutamide, a targeted AR inhibitor, has been shown to improve disease-free and overall survival rates in men with mCRPC who had previously received chemotherapy ^40^. It has also been shown to extend time to radiographic progression and death as well as improve overall survival in men with mCRPC prior to receiving chemotherapy. Delay in time to chemotherapy was also reported ^41^. Similar to enzalutamide, abiraterone was also shown to delay radiographic progression and time to chemotherapy in men with mCRPC ^42^.

In the setting of symptomatic bony metastases, radium-223 can be used. Recent data suggests that selecting the optimum combination and/or sequence of treatments may play a significant role in future responsiveness to therapies, particularly those with similar mechanisms of action ^43^.

Recent studies demonstrate that multiple factors contribute to AR reactivation and CRPC, despite castrate serum levels of androgens. Various mechanisms include changes in AR expression, structural modification through gene amplification, mutation, and alternative splicing ^44^. Therefore, agents that work via the CYP17 pathway may be required in mCRPC.

Novel agents with activity on the CYP17 pathway, such as galeterone, or modulators of AR signaling provide an alternative to abiraterone and enzalutamide in the setting of castration resistance ^45^. Ongoing research continues in the potential synergistic relationship between CYP17 inhibitors and antiandrogens ^46^. Biomarkers predictive of response or resistance may promote the best use of these treatments in the future.

Patients receiving ADT are at a greater risk for osteoporosis and bone-related complications that can significantly increase morbidity. Men with mCRPC are at an even greater risk for osteoporosis and skeletal-related events (SREs) ^47^. Prospective studies of men receiving ADT demonstrate a decrease in bone mineral density of 3% at the lumbar spine (1.4% to 3.3%) and 2% at the hip (0.7% to 3.3%) within the first year of treatment ^48^. Options for treatment-related osteoporosis thereafter include bisphosphonates, denosumab (which is a receptor activator of nuclear factor-kappaB [RANK] ligand inhibitor), and selective estrogen receptor modulators.

Zoledronic acid (Zometa), a bisphosphonate, inactivates osteoclastic activity. When compared to placebo in the setting of bony metastases and mCRPC, zoledronic acid was associated with fewer SREs at 15 months when compared with placebo (33.2% versus 44.2%; p = 0.021). Time to first SRE was improved with zoledronic acid (488 versus 321 days; p = 0.009) ^49^. Current evidence supports the monthly use of either zoledronic acid or denosumab for the reduction of SREs in men with bone-metastatic castration-resistant disease.

Denosumab is a human monoclonal antibody against nuclear factor-kappaB ligand (RANK ligand) involved in bone turnover, thus inhibiting osteoclast activity and subsequent bone breakdown. This medication was approved in 2010 for the prevention of SREs in patients with mCRPC. A randomized controlled trial demonstrated that denosumab was superior to Zometa in preventing SREs in mCRPC ^50^. The AUA guidelines recommend either medication for patients with bony metastases and castration resistance. Due to the risk of hypocalcemia with both agents, vitamin D, calcium, and frequent serum calcium monitoring is critical. Given the risk of renal insufficiency with the use of zoledronic acid, denosumab may be the preferred agent in patients with chronic kidney disease.

## Areas of future research/future directions in the field

Future directions in the field of prostate cancer management include RP for advanced and oligometastatic disease in the context of combined modality therapy ^51^ and the role of new immunotherapeutic agents, such as programmed cell death protein (PD-1) and PD-L1 inhibitors ^52, 53^. For localized disease, greater use of focal therapies such as HIFU, vapor therapy such as REZUM, and laser ablation are all areas of future research.

## Conclusion

A shift toward understanding individual tumor behavior and clinical prognostic information provides a more tailored treatment plan for patients with prostate cancer. Future directions for research include precision medicine with individualized genetic analysis and targeted therapy. These concepts represent areas of further investigation. Advances in currently available treatments translate to a wider therapeutic window, which can maximize patient benefit while minimizing morbidity.

## Abbreviations

ADT: androgen deprivation therapy

AR: androgen receptor

AS: active surveillance

AUA: American Urological Association

CaPSURE registry: Cancer of the Prostate Strategic Urologic Research Endeavor

CAPRA: Cancer of the Prostate Risk Assessment

CT: computed tomography

EBRT: external beam radiotherapy

fPSA: free PSA

HIFU: high-intensity focused ultrasound

hK2: human kallikrein 2, a prostate-specific kallikrein (protease) produced by prostate epithelium

IMPACT: Immunotherapy for Prostate Adenocarcinoma Treatment

iPSA: intact prostate-specific antigen

mCRPC: metastatic castration-resistant prostate cancer

MRI: magnetic resonance imaging

NCCN: National Comprehensive Cancer Network

PCA3: prostate cancer antigen 3

PD-1: programmed cell death protein

PET: positron emission tomography

PHI: prostate health index

PIVOT: Prostate Cancer Intervention versus Observation Trial

PSA: prostate-specific antigen

PSADT: prostate-specific antigen doubling time

PSMA: prostate-specific membrane antigen

RANK: receptor activator of nuclear factor-kappaB

RP: radical prostatectomy

SEER: Surveillance, Epidemiology, and End Results

SRE: skeletal-related events

T1c: clinical stage whereby prostate cancer is detected based on PSA value

TMPRSS-2: transmembrane protease, serine 2

tPSA: total prostate-specific antigen
